# Unconventional Stereoerror Formation Mechanisms in Nonmetallocene Propene Polymerization Systems Revealed by DFT Calculations

**DOI:** 10.1021/acs.jpca.2c04935

**Published:** 2022-09-02

**Authors:** Eugenio Romano, Peter H.M. Budzelaar, Claudio De Rosa, Giovanni Talarico

**Affiliations:** † Scuola Superiore Meridionale, Largo San Marcellino 10, 80138 Napoli, Italy; ‡ Dipartimento di Scienze Chimiche, Università degli Studi di Napoli Federico II, Via Cintia, 80126 Napoli, Italy

## Abstract

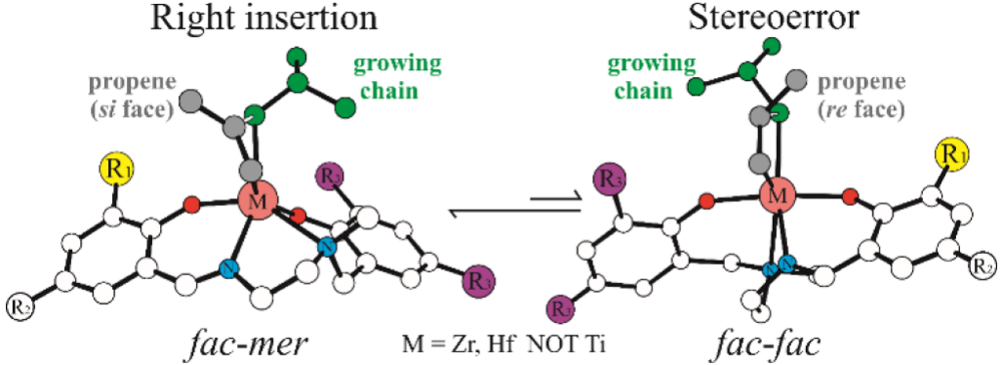

An unconventional mechanism for the stereoerror formation in propene polymerization catalyzed by *C*_1_-symmetric salalen-M systems (M = Zr, Hf) is suggested by DFT calculations. While propagation happens with the ligand in its *fac-mer* conformation, a change of ligand wrapping mode from *fac-mer* to *fac-fac* is the main source of the lower stereoselectivities obtained with Zr and Hf. This is different for the Ti analogues, where the ligand *fac*-*mer* wrapping mode does not play a role. Activation strain analysis indicates that the preference for a chain stationary mechanism of the Zr/Hf species is due to the energy required to distort the reactants (Δ*E*_Strain_) rather than to their mutual interaction (Δ*E*_Int_)_._

## Introduction

**Scheme 1:**
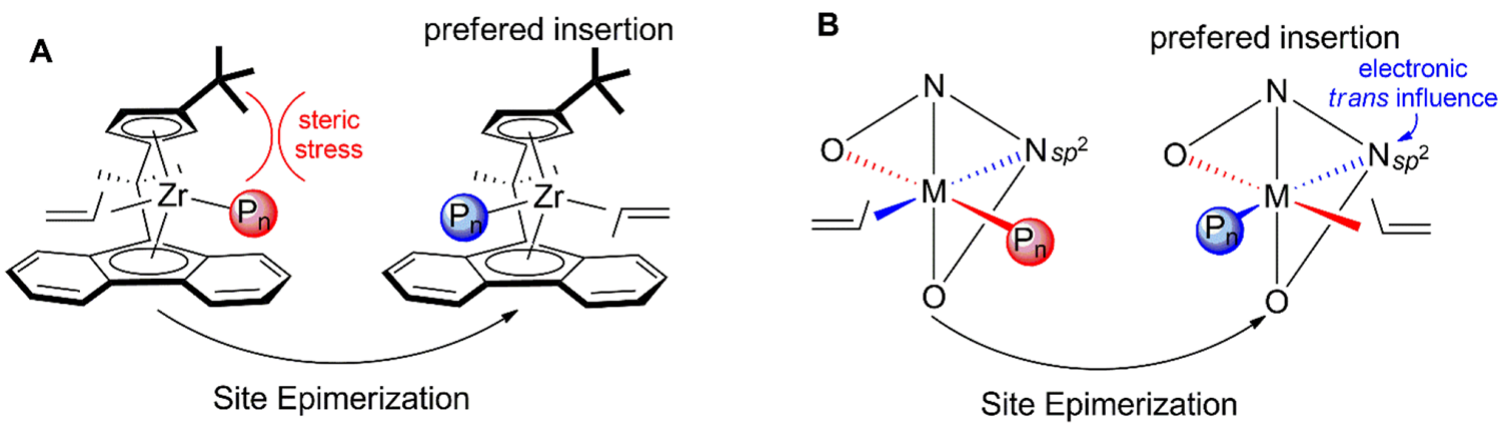
Steric and Electronic Effects for the Site Epimerization Mechanisms Proposed for *C*_1_-Symmetric Active Species Based on *ansa*-Metallocenes (A) and Salalenes (B)^17^

The study of metallocene olefin polymerization catalysts has provided a detailed understanding of polymerization mechanisms, including stereoselectivity, regioselectivity, and molar mass capability.^1,2^ Translation of these insights to new classes of catalysts has seemed straightforward, and this has in turn led to the vigorous development of new postmetallocene catalysts.^3−6^ It is often implicitly assumed that the geometry of the active site corresponds to that of the neutral catalyst precursor, although exceptions are known.^7−13^ In the case of *C*_1_-symmetric catalysts, there are two diastereotopic active sites: propagation can happen predominantly without chain backskip (“Chain Migration mechanism”, CM), predominantly with backskip after insertion (“Chain Stationary mechanism”, CS), or any intermediate situation. For metallocenes, steric factors have been used to tune this balance (see Scheme 1A).^14−16^ Interestingly, Kol et al.^17^ reported on tuning of *C*_1_-symmetric salalen complexes of Group 4, where it was proposed that the electronic asymmetry (due to the *trans* influence) was effective in enforcing a CS mechanism (see Scheme 1B). The ligand coordination mode of the active species was assumed to correspond to the *fac*-*mer* (FM) arrangement determined for the neutral catalyst precursor in the solid state.^17,18^

**Figure 1:**
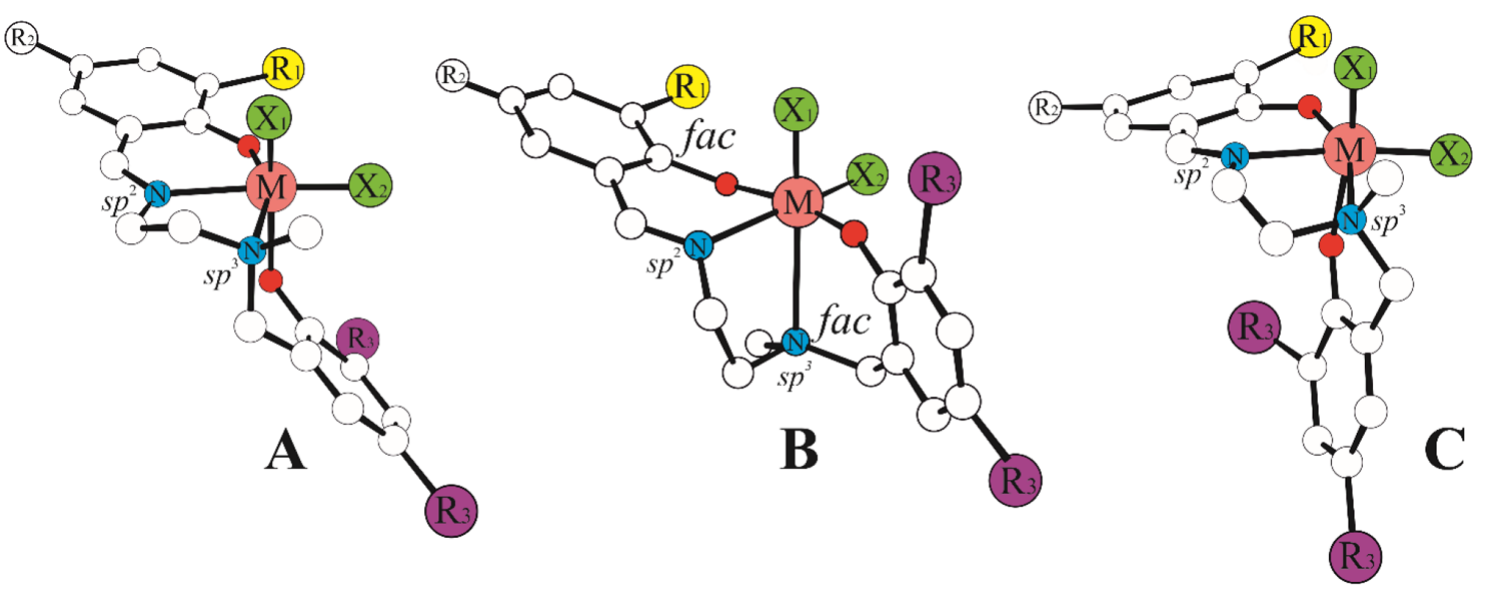
Coordination modes for salalen catalyst precursors with *fac*-*mer* (A), *fac*-*fac* (B), and a modified *fac*-*mer* ligand wrapping mode (C) reported for Ti systems.^19^

The FM coordination ensures the electronic asymmetry of the two coordination sites involved in polymerization because one coordination site is in *trans* to a phenoxy group and the other one *trans* to the sp^2^ N atom (N_imine_), see Figure 1A. However, DFT calculations indicate that the picture is more complicated: at least for Ti, the preferred wrapping mode for the cationic active species in propene polymerization is *fac*-*fac* (FF) (see Figure 1B), and the preferred FM conformation is slightly different from the one reported in the X-ray structure (see Figure 1C).^19^

**Scheme 2:**
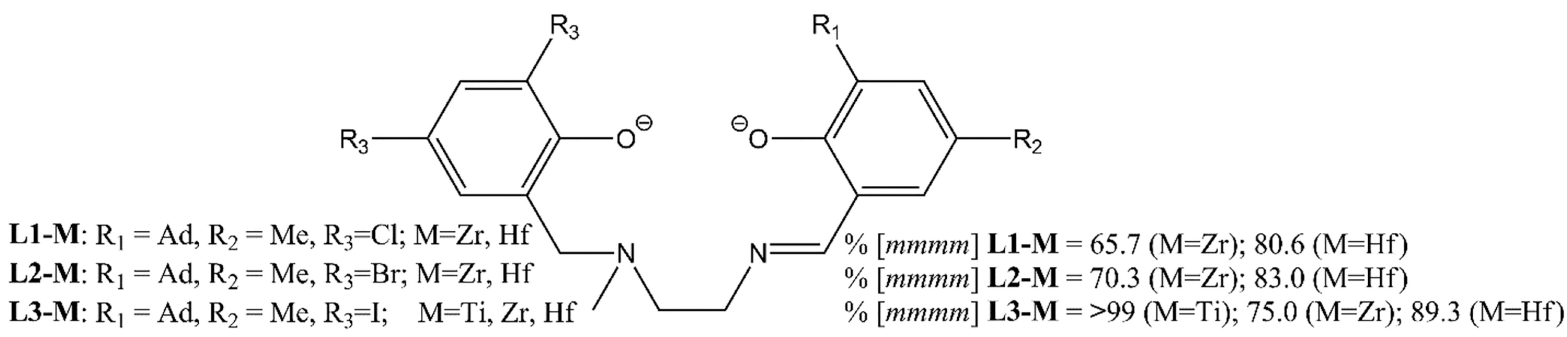
Salalen Ligands Studied in This Work (Left) and Experimental Isoselectivity for Propene Polymerization Reported as %[*mmmm*] (Right)^18^

The *trans* electronic influence is supposed to be less effective with the FF conformations, being dictated in that case by the different sp^2^/sp^3^ hybridization of the N atoms (N_imine_/N_amine_, respectively, Figure 1B), so limiting the directional site control of the chain. Understanding of the wrapping mode and hence the geometry of the active species is important not only for identifying the polymerization mechanism (e.g., chain stationary vs. chain migration) but also for successful ligand modification to enhance the stereoselectivity of the reaction. In particular, scattered and not yet explained results have been reported for the isoselectivity of propene polymerization (here considered as %[*mmmm*] detected by ^13^C NMR spectra of the polypropylenes) promoted by salalen systems combined with Group 4 metals (M = Ti, Zr, Hf) and R_1_, R_2_, and R_3_ ligand substituents (see Scheme 2).^18^ We were particularly surprised by the (large) metal effect (with Ti much more stereoselective than the Zr and Hf analogues) and by the (minimal) ligand effect of increasing the bulkiness of R_3_ substituents (going from Cl, Br to I) for Zr and Hf systems.

The latter aspect is remarkable because the R_3_ “stereodirecting role” has been proposed as the main factor for explaining the stereoselectivity of octahedral complexes (both in homogeneous and heterogeneous phases)^20−23^ (Figure S1). So, we decided to investigate in more detail the propene polymerization mechanisms of the Zr/Hf salalen catalysts shown in Scheme 2, using DFT calculations (see Computational Methods) combined with the Activation Strain Model (ASM).^24,25^ The DFT computational approach has been already tested in propene polymerization catalysis and found to be reliable,^26,27^ and in the ASM model,^24,25^ the relative energy of a molecular system is partitioned into the sum of the energies required to distort the reactants into the geometries required to react and into the strength of their mutual interaction.^28,29^

## COMPUTATIONAL METHODS

All DFT static calculations have been performed with the Gaussian09 and Gaussian16 set of programs,^30^ using the B3LYP functional of Becke and Perdew.^31,32^ The electronic configuration of the molecular systems was described with the standard split-valence basis set with a polarization function of Ahlrichs and co-workers for H, C, N, O, and Cl (SVP).^33^ Stationary points were characterized using vibrational analyses, and these analyses were also used to calculate zero-point energies and thermal (enthalpy and entropy) corrections (298.15 K, 1 bar). Improved electronic energies were obtained from single-point calculations using a TZVP basis set^34^ (SDD basis and pseudopotential^35^ at the metal and Br, I), and the SVP-level enthalpy and entropy corrections, solvation (CPCM model,^36^ toluene) and dispersion corrections^37^ (EmpiricalDispersion = D3 in the Gaussian package). The growing polymer chains were simulated by ^*i*^Bu groups and only the most stable TSs were reported for the insertion reactions. The variability of the results was also checked by using a different functional (ωB97XD)^38^ showing differences below 1 kcal/mol. For the FM species, we found two conformations mode reported in Figure 1A/C; this implies that all transition state (TS) structures reported in the paper are calculated with both conformations and the lower energies (free energies) are reported in Table 1. The ASM-Energy Decomposition Analysis (EDA) model proposed by Bickelhaupt,^24,25^ allows us to decompose the reaction energy profile into two contributions along the reaction coordinate: Δ*E* = Δ*E*_Strain_ + Δ*E*_Int_ where Δ*E*_Strain_ is the energy related to reactant deformation into the geometries required to react and Δ*E*_Int_ is the energy related to the strength of their mutual interactions. The former term is the sum of the strain related with each reactant: Δ*E*_Strain_ = Δ*E*_Strain,frag1_ + Δ*E*_Strain,frag2_ where Δ*E*_Strain,frag1_ and Δ*E*_Strain,frag2_ are the deformation energies of the fragment 1 and fragment 2 corresponding in our case to active specie plus growing polymer chain (Δ*E*_Strain(Cat)_) and the propene molecule (Δ*E*_Strain(C3H6)_), calculated with respect to minimum equilibrium geometry of the fragments. The latter term has been decomposed using the NEDA (Natural Energy Decomposition Analysis) scheme.^39^ We applied this analysis to each point obtained by IRC calculations, and Δ*E*_Strain(Cat)_ and Δ*E*_Strain(C3H6)_ have been obtained from the ASM by subtracting the energy of the minimum equilibrium geometry from the energy of the respective fragment. Simultaneously, the NEDA scheme on each point of the scan (using NBO version 7 on Gaussian16) using a TZVP basis set^34^ (SDD basis and pseudopotential at the metal)^35^ and considering only dispersion corrections^37^ has been performed, obtaining Δ*E*_Int_ and all its components (electrostatic, polarization, charge transfer, exchange, and deformation components).

**Table 1:** Electronic Energy (Gibbs Free Energy) at DFT level (kcal/mol) for Propene Insertion TSs for Complexes with FM and FF Ligand Coordination Modes

ligand–metal	FF/FM^a^	FF_1/2_^b^	FM_1/2_^b^	FF_Stereo_^c^	FM_Stereo_^d^	Calc_Stereo_^e^
**L3-Ti**	5.4 (5.3)	0.7 (0.6)	1.0 (1.6)	4.4 (4.4)	2.1 (2.3)	4.4 (4.4)
**L1-Zr**	–2.8 (−2.0)	0.8 (2.0)	5.9 (4.2)	3.0 (3.1)	3.6 (4.5)	2.8 (2.0)
**L2-Zr**	–2.3 (−1.9)	1.0 (1.8)	5.0 (4.2)	3.3 (2.7)	3.4 (4.0)	2.3 (1.9)
**L3-Zr**	–2.2 (−2.0)	1.0 (1.2)	4.8 (4.1)	3.8 (4.0)	3.4 (4.0)	2.2 (2.0)
**L1-Hf**	–2.8 (−1.6)	0.6 (1.0)	4.5 (3.8)	3.2 (3.7)	3.0 (2.6)	2.8 (1.6)
**L2-Hf**	–3.0 (−2.7)	1.5 (1.7)	5.6 (5.1)	3.0 (3.4)	3.4 (3.6)	3.0 (2.7)
**L3-Hf**	–2.6 (−2.7)	0.7 (1.3)	4.1 (4.1)	3.5 (3.9)	3.2 (4.3)	2.6 (2.7)

a Energy differences (free energies) of low-lying propene TSs at FM and FF structures (negative values indicate a preference for FM insertion.

b Energy differences (free energies) of low-lying propene TSs at site 1 and site 2 for FF and FM species (positive values indicate a preference for site 1).

c Calculated stereoselectivity at site 1 for FF structures. For definition of site 1 and site 2, see text and Figure 1.

d Calculated stereoselectivity at site 1 for FM structures. For definition of site 1 and site 2, see text and Figure 1.

e Calculated overall stereoselectivity including the contributions of all relevant ligand wrapping modes.

## Results and Discussion

Let us start the discussion by analyzing the DFT results for the propene insertion TS at the systems of Scheme 2 with M = Ti, Zr, Hf reported in Table 1. The variability of the preferred coordination mode and the existence of diastereotopic reactive sites shown in Figure 1 necessitated the calculation of 18 TSs per ligand. We label as “site 1” the olefin insertion in the position of X_1_ and the chain in the position of X_2_ (see Figure 1) for both FF and FM structures. This computational screening is summarized in Table 1, and we use the following definition for the sake of readability: (a) FF/FM (second column) is the energy difference (Gibbs free energy difference) between the lower-lying propene insertion TSs at FM and FF coordination modes, (positive values indicate a preference for FF); (b) FF_1/2_ and FM_1/2_ (third and fourth columns) report energy differences of propene TSs at sites 1 and 2 for FF and FM species, respectively, (positive values indicate a preference for site 1); (c) FF_Stereo_ and FM_Stereo_ values (fifth and sixth column) are the stereoselectivities calculated at the active species with FF and FM coordination modes; (d) Calc_Stereo_ (final column) is our computational estimate for the propene enantioface selection including the contributions of all relevant ligand wrapping modes.

**Figure 2:**
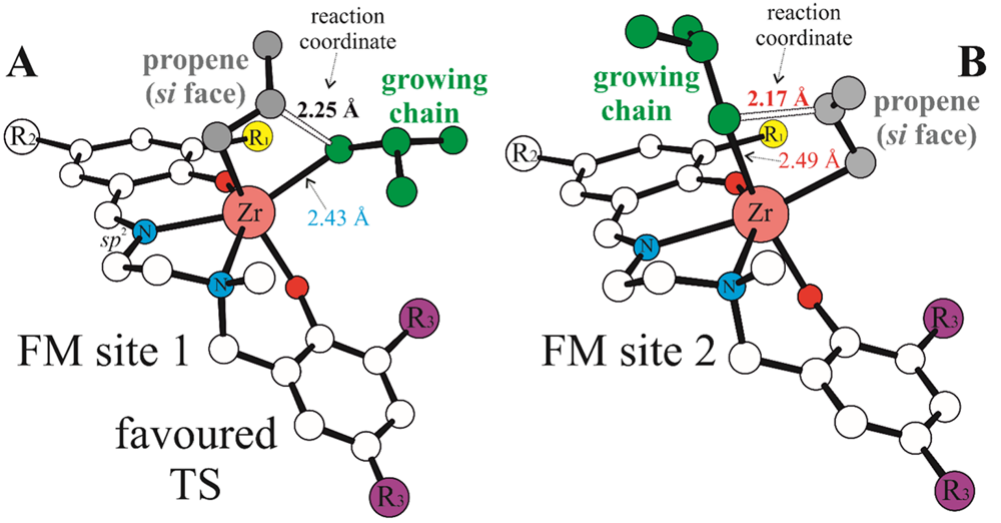
DFT optimized geometries for propene insertion (*si* face) TSs promoted by **L1-Zr** with FM ligand coordination mode: site 1 (A) and site 2 (B). H atoms omitted for clarity, distances in Å.

The first interesting result is that for Zr and Hf systems the preferred propene insertion occurs at FM geometry for **L1**-**L3** ligands. This is the opposite of what we found earlier for Ti complexes^19^ (see FF/FM results in Table 1) but is consistent with Kol’s proposal for Zr and Hf.^18^ Considering the FM_1/2_ values, the energy difference between the propene insertion at the two reaction sites (4–6 kcal/mol, see Table 1) is remarkable and similar for all three metals: this leads to an expected CS mechanism at site 1 for Zr and Hf (but not for Ti because it does not propagate through FM). The TS geometries of the preferred propene insertion at the two diastereotopic sites for **L1-Zr** system are shown in Figure 2; both sites prefer the same propene enantioface, and as expected, insertion at the site 1 having the growing chain *trans* to the N_imine_, (Figure 2A) is favored with respect to site 2 (Figure 2B).

**Figure 3:**
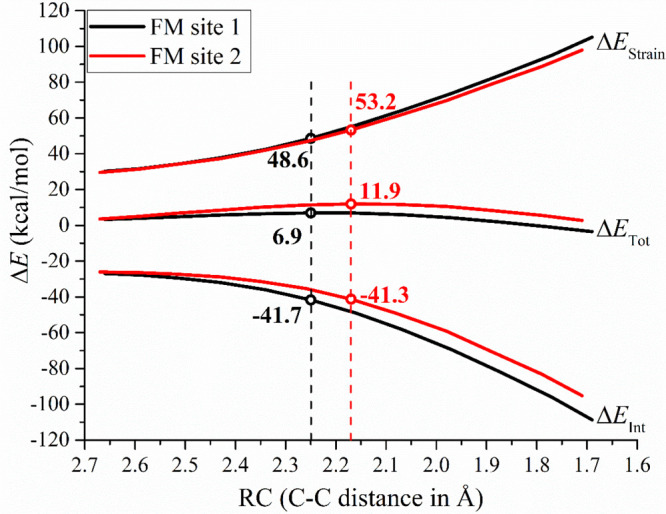
ASM analysis of the propene insertion at site 1 (black) and site 2 (red) of system **L1-Zr** in FM coordination mode as a function of the reaction coordinate. With circles are reported the values (kcal/mol) calculated at the two TS structures.

Overall, these results seem to give additional credit to the idea of Kol et al. concerning the use of electronic effects—dictated by the FM wrapping mode—to control the chain direction (Scheme 1B).^17^ Coherently, this effect is reduced to ∼1 kcal/mol for the FF geometries (see FF_1/2_ values, third column). Considering the possibility that a combination of steric *plus* electronic influence might enhance our control of polymerization, we decided to further explore this aspect using the ASM approach.^24,25^ In the insertion reaction considered here, the two fragments are the catalyst carrying the growing polymer chain and the propene molecule as described in the Computational Methods section. The Figure 3 reports the activation strain diagram of propene insertion promoted by **L1-Zr** system with the total energy (Δ*E*_Tot_) and its decomposition terms, the total strain energy (Δ*E*_Strain_) and total interaction energy (Δ*E*_Int_) against the reaction coordinate (RC) assumed as the C_C3H6_–C_chain_ distance of the forming bond (see Figure 2), with the positions of the corresponding TSs indicated by circles.

Surprisingly, we find that the main contribution to the Δ*E*_Tot_ energetic difference (5 kcal/mol, see Figure 3) is due to Δ*E*_Strain_ and not to Δ*E*_Int_ as might be expected if considering the electronic *trans* influence (Scheme 1B). This may be due, in part, to the fact that the insertion TS at site 2 occurs at a smaller distance (2.17 Å) than at site 1 (2.25 Å); thus, the olefin needs to come in closer contact with the chain, implying more deformation of both olefin and catalyst. In any case, the two terms composing Δ*E*_Strain_ (Δ*E*_Strain(Cat)_ and Δ*E*_Strain(C3H6)_) both point to a lower deformation energy of the catalyst carrying the chain and olefin at site 1 (see Table 2).

**Table 2:**
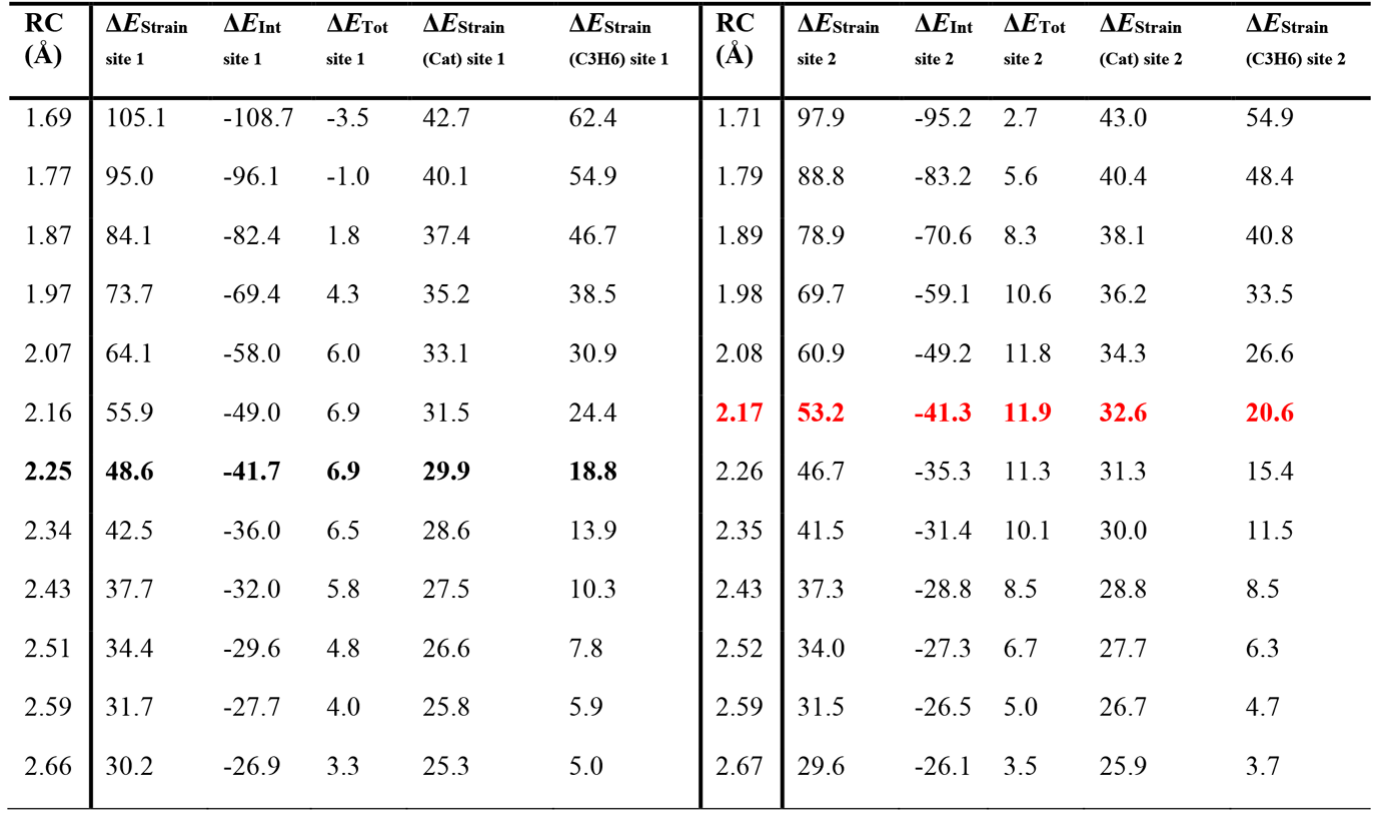
ASM Analysis for the Propene Reaction Coordinate (RC, in Å) at the Two Diastereotopic Active Sites (Site 1 on the Left and Site 2 on the Right) of **L1-Zr** Complex^a^

a In bold are reported the values obtained at the TS geometries (black for site 1 and red for site 2, respectively). Values in kcal/mol.

**Figure 4:**
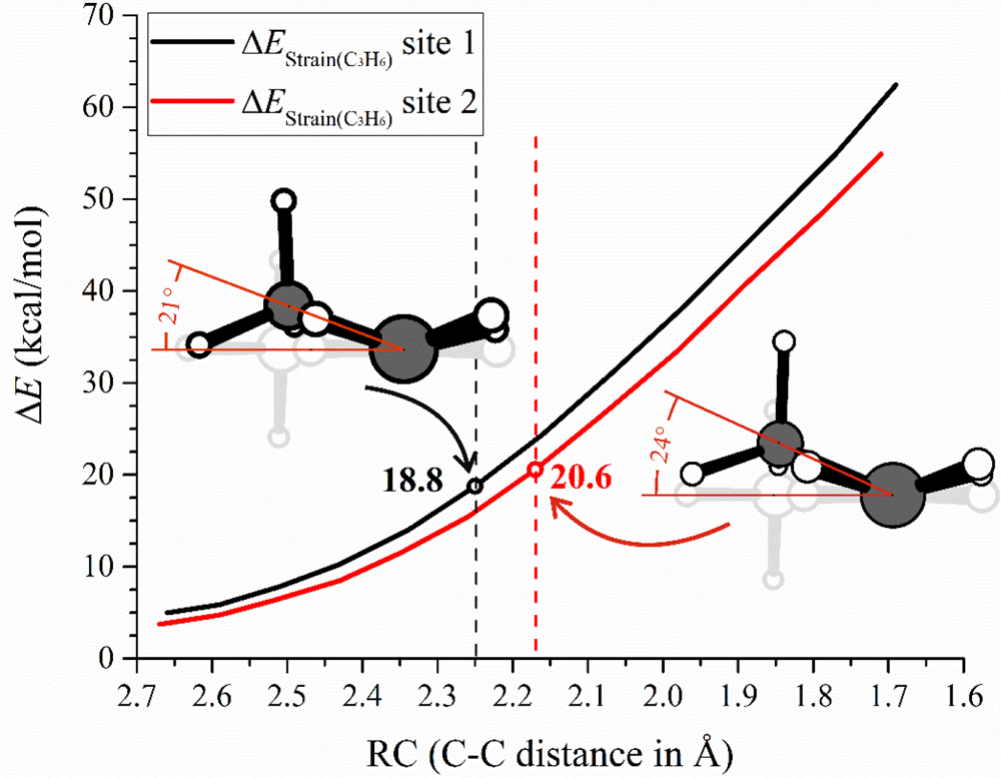
ASM analysis of the Δ*E*_Strain(C3H6)_ for propene insertion at site 1 (black) and site 2 (red) of **L1-Zr** as a function of the reaction coordinate. With circles are reported the values (kcal/mol) calculated at the TS structures. The olefin deformation is reported with respect to the free olefin (light gray).

Focusing on the olefin deformation, Figure 4 shows the Δ*E*_Strain(C3H6)_ trend for site 1 and site 2. Although the graph indicates greater olefin deformation at site 1 for equal C–C distances, by looking at the TS geometries we find that olefin deformation at site 1 costs less energy than at site 2. In Figure 4, the deformed olefin is compared to the planar free olefin with the angle in red representing the hypothetical dihedral angle H–C–C–CH_3_ that would form with the H atom in the horizontal plane. It is evident that the olefin at both TSs needs to be deformed to insert in the metal–carbon bond, but the methyl group deformation is less pronounced at site 1 than site 2.

While the difference in propagating species (Ti: FF; Zr/Hf: FM) is interesting, this does not by itself explain the lower stereoselectivity of the Zr/Hf systems.^17,18^ One could imagine that FM is intrinsically less stereoselective than FF,^40,41^ but our computational results do not support this. In fact, the FM_Stereo_ results in Table 1 show that stereoselectivity of FM insertion is even higher for Zr/Hf (>3 kcal/mol) than for Ti (2.4 kcal/mol).

**Figure 5:**
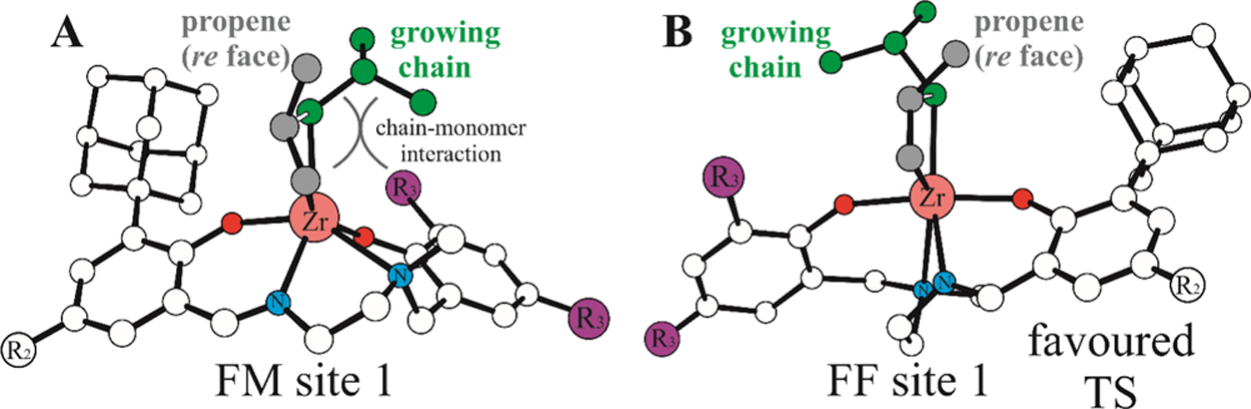
DFT optimized geometries for propene stereoerror insertion (*re* face) TSs promoted by **L1-Zr** with FM (A) and FF (B) coordination modes at site 1. H atoms omitted for clarity, R_2_ = Me; R_3_ = Cl.

The lowest TS we found for a propene stereomistake at the FM geometry is shown in Figure 5A (additional conformations with higher energies are reported in Figure S2). The bulky R_1_ substituent (adamantyl for ligands **L1**–**L3**) forces the chain to adopt a conformation *syn* to the methyl group of the propene *re* face.^20,21^ However, we find that for Zr/Hf the FF wrapping mode is accessible. Since FF insertion happens with the opposite enantioface, this turns out to be the main source of stereoerrors for Zr/Hf; Figure 5B shows the lowest FF insertion TS. In contrast, for Ti, the FM mode is not accessible and hence plays no role in stereoregulation.

In addition, the R_3_ substituents are far away from monomer and growing chain in the FF geometries (Figure 5B), which may account for the ineffectiveness of substituent bulk to tune the calculated (see Calc_Stereo_ column in Table 1) and experimental isoselectivity of Zr and Hf salalen complexes reported in Scheme 2.

To the best of our knowledge, variation of ligand wrapping mode inducing the formation of a stereoerror has not been reported in TM-catalyzed olefin polymerization. Fluxionality of active sites during the polymerization chain growth has been suggested for heterogeneous Ziegler–Natta systems^21,42,43^ as well as oscillating metallocene catalysts^44^ or bisphenoxyimine systems.^45^ However, for the first two cases, the modification of active site geometries produces stereoblock microstructures^21^ (and not an occasional stereomistake), whereas in the latter case, the interconversion between Δ and Λ forms is dictated at each insertion step.^46^

**Figure 6:**
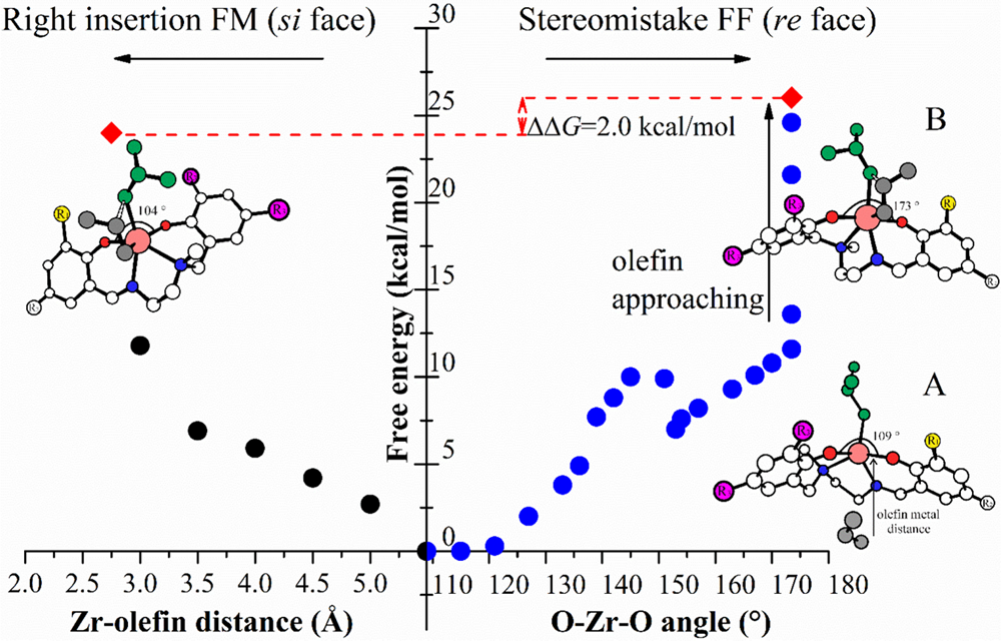
DFT calculated path of ligand O–Zr–O angle variation (from A) and further olefin capture (B) leading from penta-coordinated **LZr(P)**^**+**^ to hexacoordinate FF olefin insertions (left) and the analogous path for the FM insertion (right) where the initial O–Zr–O is already close to the one calculated at the TS geometry.

For our explanation to work, it is important that changes in ligand wrapping mode can happen easily. To test this point, we traced the path of interconversion from the pentacoordinated Zr species to the two TSs (Figure 6) on approach of the propene monomer. The pentacoordinated species (see structure A on Figure 6, right) shows the O–Zr–O angle of 109°, which is close to the one reported in the TS of the FM stereoregular insertion (Figure 6, left), whereas this angle has to increase to 173° for the FF insertion TS followed by the olefin approaching (see structure B on Figure 6, right). The results show that conformational change is possible without a significant intervening barrier, which can explain the nonblocky nature of stereoerror insertion for this system.

## Conclusions

**Scheme 3:**
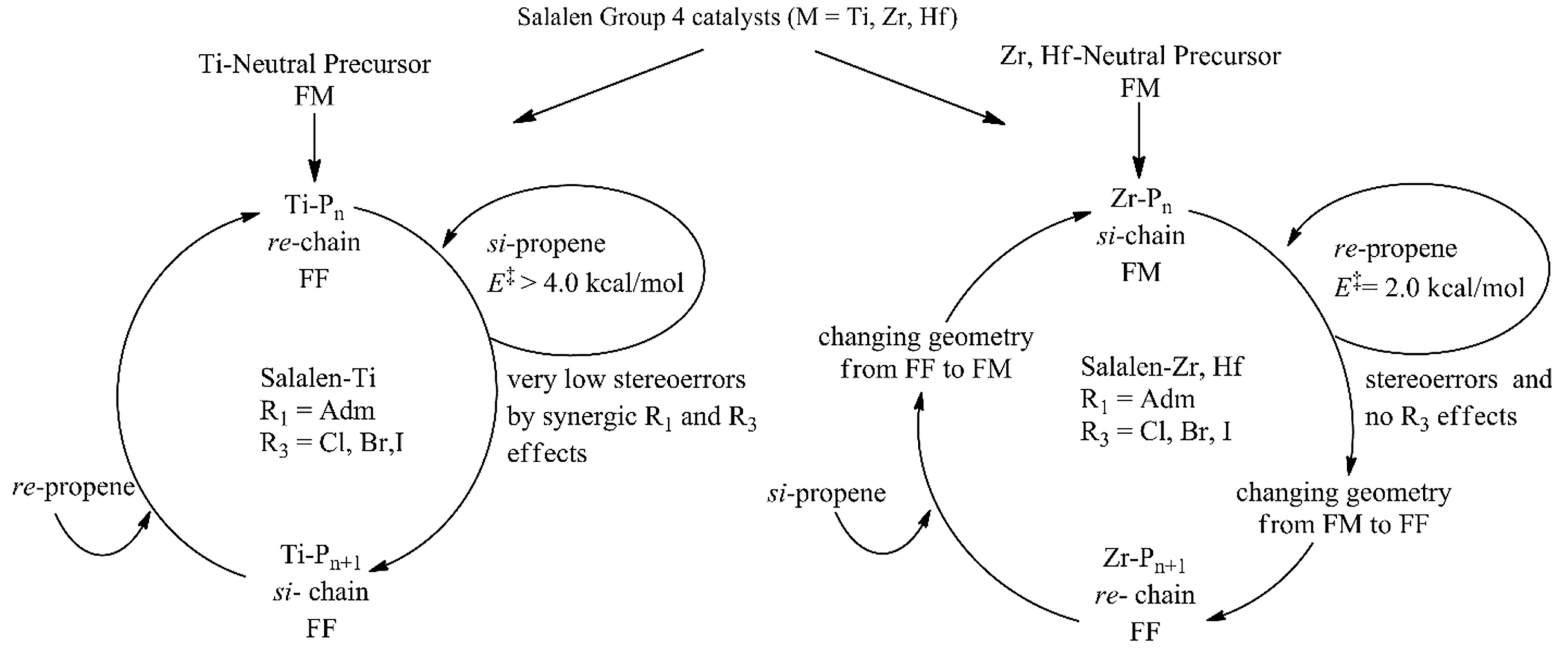
General Scheme Achieved by DFT Calculations Summarizing the Catalyst Precursor Structure, the Ligand Wrapping Mode of the Active Species, the Calculated Stereoselectivity, and the Substituent Effects on Propene Polymerization Promoted by Salalen-Ligand with M = Ti, Zr, Hf

The above considerations are summarized in different catalytic cycles proposed for Ti and Zr/Hf salalen systems (Scheme 3).

The Ti active species features a ligand wrapping mode (FF) that differs from its neutral precursor (FM) but is otherwise “normal” in its mechanism of stereoerror formation. In contrast, the Zr and Hf active species propagate as FM but generate stereoerrors via easy formation of an FF-wrapped mode. The easy accessibility of the Zr FF wrapping mode explains the formation of isolated stereoerrors, differing sharply from cases of “oscillating” catalysts where active species interconversion is slow on the propagation time scale.^47,48^ Thus, active species fluxionality (and/or change in the wrapping mode) is yet another factor to keep in mind when tuning the polypropylene microstructure analogously to the peculiar chiral recognition recently reported on the stereocontrolled ring-opening polymerization of lactide.^49−51^

## Supporting Information Available

Figures S1 and S2 (PDF)
Cartesian coordinates of the structures discussed in the text (XYZ)

## Supplementary Material

jp2c04935_si_001.pdf

jp2c04935_si_002.xyz
