# Supplementary material for: Unconventional Stereoerror Formation Mechanisms in Nonmetallocene Propene Polymerization Systems Revealed by DFT Calculations
Source: J Phys Chem A. 2022 Sep 2;126(36):6203–9. doi: 10.1021/acs.jpca.2c04935 (PMC9483984; doi:10.1021/acs.jpca.2c04935)
Supplement: Supplementary file 1 — jp2c04935_si_001.pdf [file jp2c04935_si_001.pdf]

SUPPORTING INFORMATION

Unconventional Stereoerror Formation Mechanisms in Non-Metallocene Propene Polymerization Systems Revealed by DFT Calculations

Eugenio Romano,[a] Peter H.M. Budzelaar,[b] Claudio De Rosa,[b] Giovanni Talarico*[a,b]

[a]Scuola Superiore Meridionale, Largo San Marcellino 10, 80138 Napoli, Italy
[b]Dipartimento di Scienze Chimiche, Università degli Studi di Napoli Federico II, Via Cintia, 80126 Napoli, Italy

1. **Stereodirecting role of R_3 substituents: Figure S1 Page S2**
2. **Additional source of stereoerrors: Figure S2 Page S3**

1. Stereodirecting role of R_3 substituents:

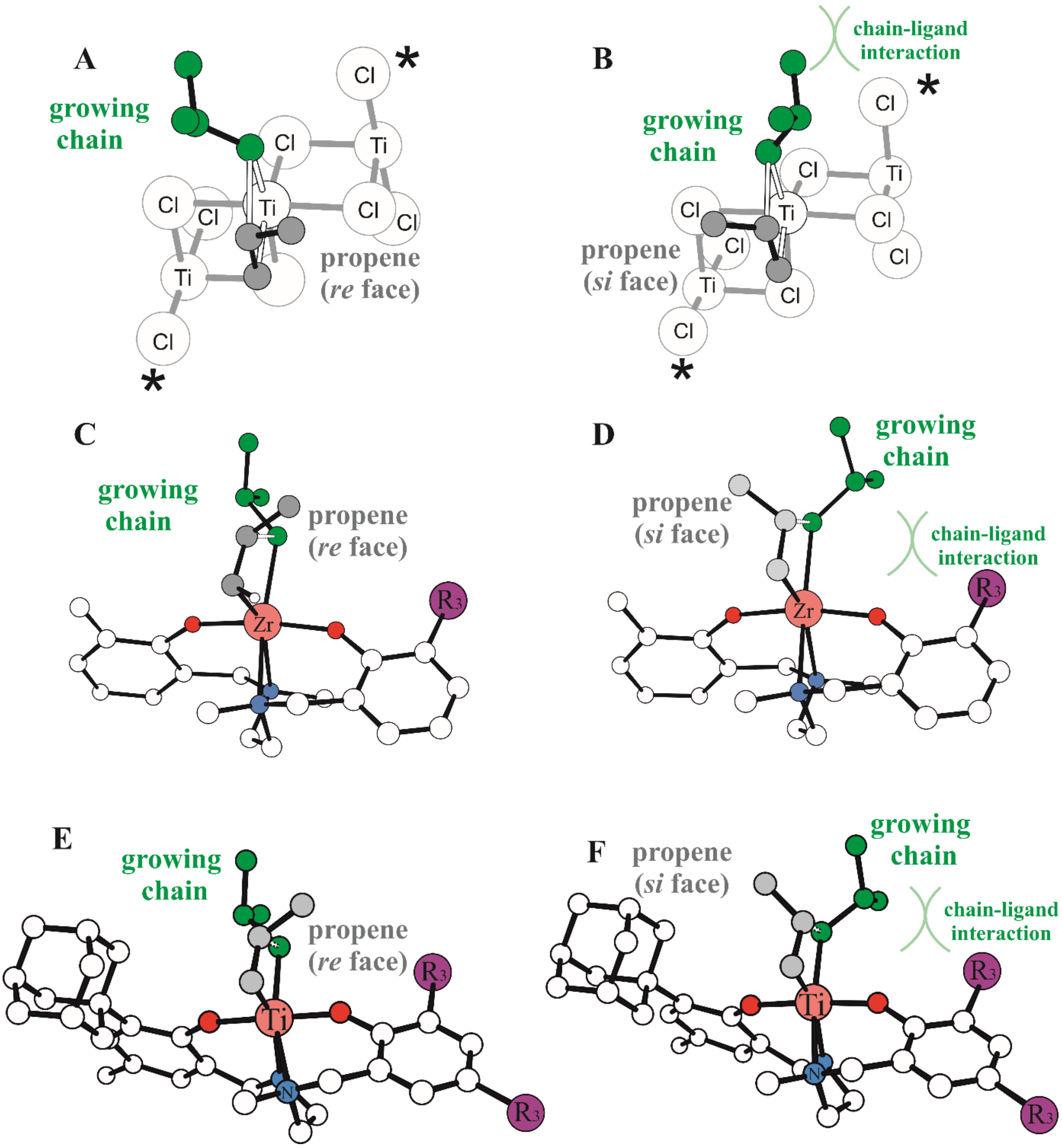

Figure S1. Stereodirecting role of R_3 substituent to orient the growing polymer chain, which, in turn, selects the monomer enantioface for a model of heterogeneous Ziegler-Natta system (**A**,**B**), for salan-Zr complexes (**C**,**D**) and salalen-Ti catalysts (**E**,**F**).

.

2. Additional source of stereoerrors:

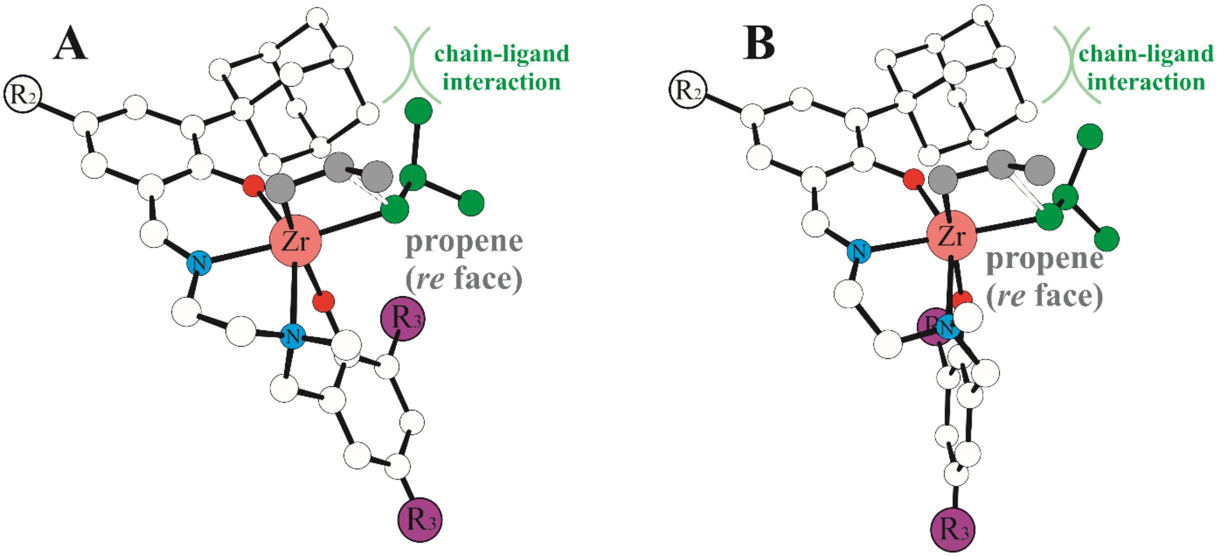

Figure S2. Additional sources of stereoerror TSs revealed by DFT calculations at the FM structures. These TSs are higher in energy with respect to the ones reported in the Figure 5 of the main text.
